# Million Migrants study of healthcare and mortality outcomes in non-EU migrants and refugees to England: Analysis protocol for a linked population-based cohort study of 1.5 million migrants

**DOI:** 10.12688/wellcomeopenres.15007.1

**Published:** 2019-01-17

**Authors:** Rachel Burns, Neha Pathak, Ines Campos-Matos, Dominik Zenner, Srinivasa Vittal Katikireddi, Morris C. Muzyamba, J. Jaime Miranda, Ruth Gilbert, Harry Rutter, Lucy Jones, Elizabeth Williamson, Andrew C. Hayward, Liam Smeeth, Ibrahim Abubakar, Harry Hemingway, Robert W. Aldridge

**Affiliations:** 1 Centre for Public Health Data Science, University College London, London, UK; 2 Institute of Epidemiology and Healthcare, University College London, London, UK; 3 Public Health England, London, UK; 4 Migration Health Division, International Organization for Migration, Brussels, Belgium; 5 Institute for Global Health, University College London, London, UK; 6 MRC/CSO Social and Public Health Sciences Unit, Institute of Health and Wellbeing, College of Medical, Veterinary and Life Sciences, University of Glasgow, Glasgow, UK; 7 CRONICAS Center of Excellence in Chronic Diseases, Universidad Peruana Cayetano Heredia, Lima, Peru; 8 School of Medicine, Universidad Peruana Cayetano Heredia, Lima, Peru; 9 Administrative Data Research Centre for England, University College London, London, UK; 10 Faculty of Humanities and Social Sciences, University of Bath, Bath, UK; 11 UK programme manager, Doctors of the World, London, UK; 12 Faculty of Epidemiology & Population Health, London School of Hygiene and Tropical Medicine, London, UK; 13 Department of Non-Communicable Diseases Epidemiology, London School of Hygiene and Tropical Medicine, London, UK; 14 Institute of Health Informatics Research, Faculty of Population Health Sciences, University College London, London, UK

**Keywords:** migration, migrant, refugee, health, healthcare, mortality

## Abstract

**Background:** In 2017, 15.6% of the people living in England were born abroad, yet we have a limited understanding of their use of health services and subsequent health conditions. This linked population-based cohort study aims to describe the hospital-based healthcare and mortality outcomes of 1.5 million non-European Union (EU) migrants and refugees in England.

**Methods and analysis: **We will link four data sources: first, non-EU migrant tuberculosis pre-entry screening data; second, refugee pre-entry health assessment data; third, national hospital episode statistics; and fourth, Office of National Statistics death records. Using this linked dataset, we will then generate a population-based cohort to examine hospital-based events and mortality outcomes in England between Jan 1, 2006, and Dec 31, 2017. We will compare outcomes across three groups in our analyses: 1) non-EU international migrants, 2) refugees, and 3) general population of England.

**Ethics and dissemination: **We will obtain approval to use unconsented patient identifiable data from the Secretary of State for Health through the Confidentiality Advisory Group and the National Health Service Research Ethics Committee. After data linkage, we will destroy identifying data and undertake all analyses using the pseudonymised dataset. The results will provide policy makers and civil society with detailed information about the health needs of non-EU international migrants and refugees in England.

## Introduction

In 2017, 8.6 million (15.6%) people living in England were born abroad, with 5.3 million (9.2%) born outside of the European Union (EU) ^1^. However, little is known about how these international migrants use England’s National Health Service (NHS) or their subsequent health needs and mortality outcomes. Here we define international migrants as people born outside of England ^2^. This may include, for example, people who either have chosen to migrate (e.g. work, study, or join families) or those who may have been forced to migrate due to conflict, persecution or environmental disasters (e.g. refugees and asylum seekers).

A previous systematic review of healthcare usage in Europe showed that usage of accident and emergency (A&E) services by international migrants was high, but that screening and outpatient care usage was low ^3^. Similarly, a recent study from Scotland showed that some ethnic minorities, whether migrants or their offspring, have higher levels of avoidable hospital admissions when compared to the non-migrant white Scottish population ^4^. These findings suggest that migrants may be receiving poor quality primary and preventative healthcare or face barriers to accessing health services.

In England, no large scale studies have been conducted to date that were able to examine migrants’ usage of hospital-based healthcare services. One primary care based study in England found that individuals who registered with a general practitioner (GP) for the first time when over the age of 15 - used as a proxy for international migrants - had half the rate of hospital admissions than the general population ^5^. However, another study found that only one-third of new migrants had registered with a GP, complicating the use of this proxy measure as well as highlighting poor uptake of primary healthcare registration ^6^. Other studies in England ^7, 8^ have used country of birth to examine migration, but did not have information on the date of migration, the country which an individual was migrating from, or the visa category under which they entered England. These studies illustrate the methodological challenges of accurately identifying migrants within national data sources and the lack of available information on their migratory history, thus limiting the interpretations of previous research.

Despite poor access to preventative healthcare, there is evidence that migrants have a mortality advantage compared to host populations in the high-income countries to which they migrate. We recently conducted a systematic review and meta-analysis on the global patterns of mortality data in international migrants ^9^. Our review showed that the levels of mortality in international migrants, measured using standardised mortality ratios, were lower compared to the host population in the countries of destination for most disease causes. Two exceptions to these findings were an increase in mortality due to infectious disease and external causes of mortality ^9^. As we found very little data on refugees, asylum seekers and other forced migrants, our data is most representative of international migrants in high-income countries who are studying, working or have joined family members. Although our findings supported the idea of the healthy migrant hypothesis - an empirically observed mortality advantage of migrants relative to the host population ^10^, there is evidence from migrants residing in England and Wales that suggests that this advantage declines with age ^7, 8^. However, these studies were not able to assess whether this advantage also changed with duration of residence in England.

The evaluation of morbidity and mortality outcomes of migrants has been limited by difficulties in their identification in national data sources. To date, we have no data in England linking migrant and refugee health service usage and subsequent health conditions and mortality outcomes. There are existing disparate data sources on the health of international migrants; however, despite their existence, these data have not been integrated or analysed systematically. These data contain information that would enable us to comprehensively evaluate the health needs in migrants and refugees and develop evidence on how to improve access to hospital-based health services and preventative healthcare in England. The Million Migrant study will generate this evidence for the first time by linking records that contain data on the health outcomes for 1.5 million non-EU migrants and refugees.

## Aim and objectives

**Figure 1.**
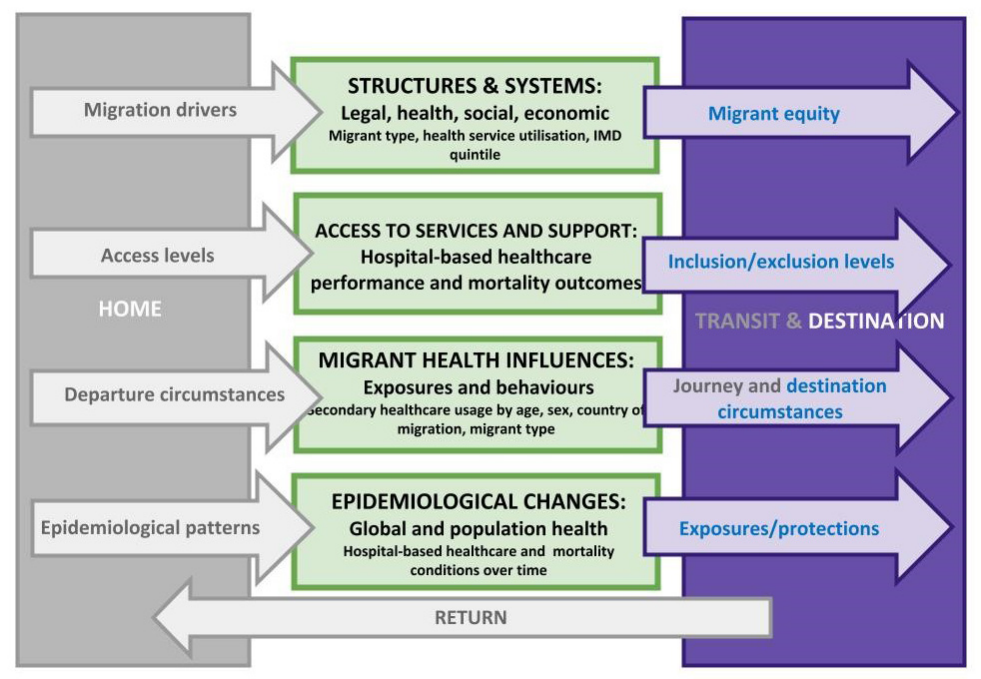
Conceptual framework for influences on migration and health adapted from UCL-Lancet Commission on Migration and Health ^12^. * The boxes and words in grey will not be measured directly in the Million Migrant study.

The Million Migrant study will be a population-based cohort study that aims to examine secondary healthcare performance (e.g. quality and accessibility) and mortality in 1.5 million non-EU migrants and refugees in England. There are two main objectives of the study. First, to profile hospital-based healthcare performance by identifying existing health conditions and examining hospital admissions, readmissions and duration of admission of non-EU migrants and refugees compared to the general population in England. Second, to investigate mortality outcomes by health condition for non-EU migrants and refugees in comparison to the general population. This objective will examine whether or not our data replicates the mortality advantage of international migrants found in the literature. The study includes analyses that acknowledge the wider determinants influencing the health of international migrants such as the legal, social, economic and health structures and systems, health service access and support, exposures and behaviours, and epidemiological changes associated with population mobility ( Figure 1).

## Protocol

### Data collection, processing, and linkage

**Figure 2.**
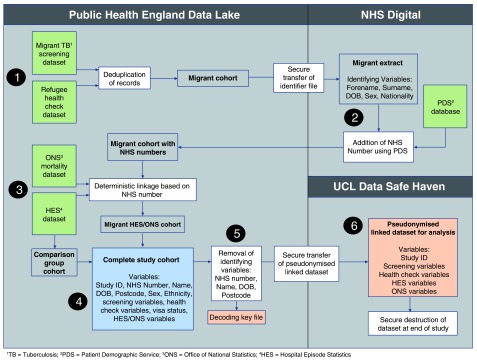
Study data flows.

Our study will link four data sources as outlined in Figure 2. First, non-EU migrant tuberculosis pre-entry screening data. This data set contain records on international non-EU migrants resident in a country where tuberculosis is common (40 cases per 100,000 people), and who are planning to come and live in United Kingdom (UK) for more than 6 months ^11^. Individuals in this dataset were screened by the UK pre-entry tuberculosis screening programme between Jan 1, 2006, and Dec 31, 2017. UK pre-entry tuberculosis screening was conducted either by the International Organization for Migration (IOM) or by international clinics recognised by the UK Home Office and quality assessed by Public Health England (PHE). Second, refugee pre-entry health assessment. Refugees undergo a health assessment that allows pre-departure information to be shared with local authorities and health services in the UK. Refugee health assessments were conducted by IOM between Mar 1, 2013 and Dec 31, 2017. Our study will therefore include two cohorts of international migrants to the UK - non-EU migrants and refugees that will be linked to the final two datasets from Jan 1, 2006, and Dec 31, 2017. Third, national hospital episode statistics (HES), including hospital admissions and attendances. Fourth, Office of National Statistics (ONS) death records which contain cause of death information on all deaths between Jan 1, 2006, and Dec 31, 2017. We focus on non-EU migrants because of their availability in this dataset, but also because EU migrants are more likely to dual-use health systems and less likely to need adaptation in terms of cultural competence ^13^. The analysis will be limited to England as the HES data will only be obtainable for English hospitals.

We will obtain identifying variables (forename, surname, aliases, date of birth, sex, country of origin, country of departure, date of tuberculosis pre-entry screening or refugee pre-entry health assessment, and visa category) from all non-EU migrants and refugees under appropriate legal and ethical approvals. We will then use the Personal Demographics Service (PDS), the national electronic database of NHS patient information such as name, address, and date of birth, to identify and add NHS numbers by matching on these identifying variables to the tuberculous pre-entry screening and refugee health assessment records. Where available, NHS numbers that have already been added by PHE will be integrated. Once the non-EU migrants and refugees have been matched to their NHS number, we will then undertake deterministic linkage using NHS number to identify HES and ONS mortality records. Personal identifiers will then be removed, resulting in a pseudonymised linked dataset which will be securely transferred to UCL for data analysis, as outlined in Figure 2. We will compare matching levels (e.g. whether an individual record is linked to their NHS number or not) across different age, sex, year and age of entry to UK and migrant country of origin groups. The non-EU migrant tuberculosis pre-entry screening dataset might contain multiple duplicate records for some individuals who require repeat tuberculosis screening. Duplicate records will be analysed on the basis of whether they occurred within 12 months of each other or not as per previously defined rules ^14^ provided in full in Extended data file 7. Non-EU migrant and refugee data are cleaned by the IOM epidemiology unit in coordination with clinics to ensure that records included all results on individuals screened and that any duplicate entries resulting from administrative error were removed or consolidated into one record. Cleaning and consistency checking of the final dataset will be undertaken by examining the distribution of variables, the range of individual variables, and missing data.

### Ethics and information governance

To undertake this work, we require access to patient identifiable data without individual consent. We will apply to the Secretary of State for Health through the Confidentiality Advisory Group (CAG) to obtain approval for this work. After data linkage (see earlier section) we will destroy all identifying data and undertake all analyses using the pseudonymised dataset outlined in Figure 2. We will also seek ethical approval for the study from an NHS Research Ethics Committee. We believe that the benefits of this study for the migrant population outweigh any risks. The primary risk that could be anticipated is a data breach of sensitive information; we are developing a comprehensive data management and data sharing plan to minimise this such that no risks are anticipated. The dataset will be created and analysed by a team with extensive experience of handling large datasets securely, and the organisations involved in this work (UCL and PHE) have extensive Information Security and Governance procedures in place to minimise this risk.

### Comparator groups

We will compare three groups in our analyses: 1) non-EU international migrants, 2) refugees, and 3) general population in England. We aim to disaggregate the non-EU migrants and refugees by: 1) age at migration, 2) sex, 3) ethnicity, 4) visa category, 5) country of origin, and 6) date of screening. We will examine each group over the follow up time period. For refugees, we will also examine country of departure as it is often different from the country of origin. Due to their smaller numbers, we will likely examine refugees by World Health Organisation (WHO) region of origin instead of country they migrated from. Final geographical categorisations used for migrant groups will be taken to minimise risk of disclosure and therefore migrants may be grouped into WHO sub-regions. Secondly, we will compare the non-EU migrants and refugees to the general population in England by deprivation level (Index of Multiple Deprivation - IMD - quintile). This will be done using an anonymous HES sample. Here the general population will be composed of mostly England-born residents, along with other types of international migrants who did not partake in one of the two screening programmes. These include EU migrants, international migrants that are not required to get a pre-entry tuberculous screening (e.g. migrants from low-tuberculous countries such as the United States), irregular migrants (e.g. undocumented), and migrants and refugees who arrived before the start of their subsequent screening programmes.

### Outcomes

We present a series of outcomes for our healthcare and mortality analyses. We chose outcomes to ensure our analysis is consistent with those used in previous published analyses ^4, 7, 8^, in addition to outcomes that are of high interest to researchers but that previous studies were not powered to collect. Moreover, we have chosen a range of outcomes that collectively reflect the priorities of health policy makers as well as migrants and refugees who attended our patient engagement workshops on consent process, data linkage and analysis.

***Hospital-based healthcare outcomes.*** We will profile the following hospital-based healthcare outcomes in non-EU migrants and refugees: 1) hospital attendances (inpatient, outpatient, and A&E), 2) hospital admissions (inpatient), 3) duration of hospital admission, and 4) 30 day emergency readmissions. We will explore these four outcomes by sub-conditions where appropriate. The clinical definitions and methodological approaches for each outcome are provided in Table 1. Full clinical definitions of each outcome’s subgroup are provided in Table 2. HES currently uses ICD-10, the 10th version of the international classification of diseases and related health problems, to code for conditions and OPCS-4, the classification of interventions and procedures, to code all interventions and procedures. ICD-10 and OPCS-4 code lists for each are provided in Extended data file 2– 5.

**Table 1.** Clinical definition and methodological approach for hospital-based healthcare and mortality outcomes

Outcome	Clinical Definition	Statistical Definition	Example Statistical Modelling Approach *
**Hospital-based healthcare outcomes**			
Hospital attendances	Number of hospital attendances in inpatient, outpatient, or A&E.	Binary indicator for attendance with specific condition (yes/no). Time to event defined as entry into cohort date until end of follow up.	Cox proportional hazards model
Hospital admissions	Admission into the hospital as an inpatient.	Binary indicator for specific admission (yes/no). Time to event defined as entry into cohort index attendance until the end of follow up.	Cox proportional hazards model
Duration of hospital admission	Number of days spent in hospital as an inpatient.	Numerical indicator for number of days.	Cox proportional hazards model
30 day emergency readmissions	Emergency admissions to any hospital in England occurring within 30 days of the last, previous discharge from hospital after admission.	Binary indicator for emergency readmission (yes/no) recorded within 30 days of the index admission discharge date. Emergency admissions are defined as those where the admission method is waiting list, booked or planned (11, 12 or 13). To be explored in subgroup of people with an initial hospitalisation.	Logistic regression
**Mortality outcomes**			
Death from all causes	Deaths in England from any cause.	Binary indicator for death due to a all-causes (yes/no). Deaths will primarily be identified through linkage to ONS deaths registration data, but also through HES (where the Method of Discharge field is coded as “dead” (4)) as the latter method may better ascertain information on recent deaths where there is a delay in death registration (e.g. because a coroner’s report is required).	Calculation of SMR using ONS death data by age and gender; rates, life expectancy
Death due to a specific condition	Death due to one of the subgroup conditions.	Binary indicator for death due to a specific condition (yes/no). Time to event defined as entry into cohort index attendance until death.	Cox proportional hazards model; calculation of SMR using ONS death data by age and gender; rates, life expectancy

*final statistical modelling approach to be confirmed during analysis stage after review of data and modelling assumptions.

***Mortality outcomes.*** We will examine the following mortality outcomes in non-EU migrants and refugees: 1) death and 2) death due to a specific condition. The clinical definition and the methodological approaches for each outcome are provided in Table 1. Full clinical definitions of each outcome’s condition are provided in Table 2. ONS datasets use ICD-10 codes to code for the health condition registered at death. ICD-10 code lists for each are provided in Extended data file 2– Extended data file 5. Lastly, we aim to examine if the mortality advantage found in the literature can be replicated with this migrant and refugee population in England.

**Table 2.** Clinical definition for hospital-based healthcare and mortality subgroup outcomes. Extended data can be found at DOI 10.17605/OSF.IO/FUTS4 ^15^.

Outcome Subgroups	Clinical Definition
Ambulatory care sensitive (ACS) conditions ( Extended data file 4)	Conditions where effective community care can prevent inpatient hospital admission or death.
Amenable conditions	Conditions where hospital admissions or death could be avoided through high quality preventative healthcare ^16^.
Preventable conditions	Conditions where all or most hospital admissions or deaths from a specific cause could be avoided by established medical or public health interventions ^16^.
Avoidable conditions ( Extended data file 2 and Extended data file 3)	Conditions that are considered preventable, amendable or both, where each admission or death is only counted once. When cause of admissions or death falls within both the preventable and amenable definition, all admissions or deaths from that cause are counted in both categories when they are presented separately ^16^.
Sexual and reproductive health conditions and treatments ( Extended data file 5)	Conditions where hospital admissions or death is for a sexual and reproductive health outcome or treatment. These are split into seven domains based on the Guttmacher-Lancet commission on sexual and reproductive healthcare and rights: abortion, contraception, human immunodeficiency virus and other sexually transmitted infections, maternal and neonatal health, fertility, gender-based violence and reproductive cancers ^17^.
Tuberculosis, HIV, Hepatitis B & C ( Extended data file 2 and Extended data file 3)	Conditions where hospital admissions or death is for tuberculosis, HIV, Hepatitis B or Hepatitis C.
Common mental and behavioural disorders ( Extended data file 6)	Conditions where hospital admissions or death is for a common mental and behavioural disorder.
Multimorbidity	Co-existence of two or more chronic conditions, each one of which is either: (1) a physical non- communicabledisease of long duration, such as a cardiovascular disease or cancer; (2) a mental health condition of long duration, such as a mood disorder or dementia; or (3) an infectious disease of long duration, such as HIV or hepatitis C ^18^.
All causes *	Death due to any cause.
ICD-10 chapter * ^19^	Death due to a specific conditions, such as infectious disease, disease of the blood, cardiovascular diseases, digestive disease, genitourinary disease, musculoskeletal disease, nervous disease, respiratory disease, endocrine disease, injury or external causes, mental and behavioural, or neoplasms ^20^.
Maternal deaths *	Death of a woman while pregnant or within 42 days of termination of pregnancy, irrespective of the duration and site of the pregnancy, from any cause related to or aggravated by the pregnancy or its management but not from accidental or incidental causes ^21^.

*Subgroup only applies to the mortality outcomes.

### Entry and exit from cohort

We will include all visa applicants (non-EU migrants) from 101 countries (see Extended data file 1) with pre-entry tuberculosis screening in this study and all refugees who underwent pre-entry health assessment.

Individuals will enter the cohort at whichever is the latest of: date at which they were screened for tuberculosis pre-entry screening or refugee pre-entry health assessment. Individuals will be followed up until the earliest of: end of the follow-up period (31st December 2018), emigration, or death. Individuals found to have tuberculosis at pre-entry screening will not enter the cohort as they are not given a certificate of clearance for tuberculosis and therefore their visa process is put on hold until they are treated and a certificate is then produced. Only then will they enter the cohort.

Individuals in our cohort will be at risk of hospital admission or death from the date of entry until the first of the following events: death, emigration, or the end of the follow-up period. Since data were unavailable to indicate whether an individual migrant is living in Scotland/Wales/Northern Ireland (or resettlement to these countries in the case of refugees), or emigration, these events will be accounted for probabilistically by multiple imputation and building on previously described methods ^14, 22^.

### Sample size

1,700,000 non-EU migrants who underwent pre-entry tuberculous screening to enter the UK between 2005-2017 will be included. After removal of duplicate records there will be just over 1,500,000 unique individuals. Approximately 10% of migrants move to Scotland and Northern Ireland and will not be linked to HES and ONS data. Therefore, linkage to HES and ONS will be on approximately 1,380,000 non-EU migrants.

Table 3 provides examples of the precision by which prevalence estimates will be estimated and the changes in relative risk detectable between migrant subgroups. The study therefore has sufficient statistical power (80%) to detect changes in common outcomes (e.g. admission due to ambulatory care sensitive condition) and rare outcomes (e.g. mood (affective) disorders), at the 5% significance level.

**Table 3.** Sample size calculations that illustrate the 95%CIs with which the rates for common (ACS admission) and uncommon (Mood (affective) outcomes can be estimated for migrant subgroups, and the difference between these migrant subgroup rates that can be detected assuming type 1 error = 0.05 and power = 0.8.

	Outcome/risk factor			
Example	Overall rate (95%CIs) per 1000 person years at risk	Subgroup size	95%CIs of rate *	Hazard ratio reduction detectable **
**Admissions due to ambulatory** **care sensitive (ACS) conditions**	8.4 (8.4-8.4) ^23^	5000 (e.g. Cambodian migrants)	6.1-11.3	0.64
		10000 (e.g. Kenya)	6.7-10.4	0.73
		100000 (e.g. Bangladesh)	7.8-9.0	1.0 *
**Mood (affective) disorders**	0.42 (0.42-0.43) ^24^	5000	-	-
		10000	0.0-1.0	0.3
		100000	0.38-0.46	1.0 *

*This column indicates the confidence interval for the subgroup assuming that the subgroup rate is the same as the general population overall rate (e.g. 8.4 per 1,000 for ACS conditions). **This column indicates the hazard ratio detectable for the subgroup using the largest group as baseline (e.g. 100,000 Bangladesh) and assuming this baseline group has the same rate as the overall population level (e.g. 8.4 per 1,000 for ACS conditions).

### Analysis plan

Our analysis plan has been designed to meet our two main objectives of profiling hospital-based healthcare and mortality outcomes for non-EU migrants and refugees. To achieve this, we will undertake the analysis in three phases. In the first phase we will summarise and compare baseline characteristics (see Table 4) between the non-EU migrant group, the refugee group and the general population in England. With the exception of ethnicity, all baseline characteristics are anticipated to be fully recorded (chronic disease is presumed to be absent unless recorded). Missing values for ethnicity will be analysed grouped as “not recorded”. In the second phase, we will summarise the hospital-based healthcare and mortality outcomes of the three study populations, using the general population of England as a reference. We will estimate the crude association between each of the outcomes and these study population groups. We will then re-estimate the association between each of the outcomes and the study population group after adjusting for characteristics at the time of hospital admission or time of death: age, sex, chronic disease, calendar time period, and reason for hospital admission or cause of death. Finally, an appropriate statistical model (selected on the basis of meeting assumptions such as proportional hazards for Cox regression) will be used to analyse the relationship between the study comparison groups and each of the outcomes. Crude models will be fitted prior to adjustment for “baseline” measurements at or before the index admission. We will write-up the analysis in accordance with the Reporting of studies Conducted using Observational Routinely-collected Data (RECORD) statement ^25^.

**Table 4.** Patient characteristics in the time prior to the index admission will be collated as baseline measurements.

Variable	Description
Age at migration	(in years) as recorded at the non-EU migrant pre-entry tuberculous screening or refugee pre-entry health assessment
Sex	as recorded at the non-EU migrant pre-entry tuberculous screening or refugee pre-entry health assessment
Ethnicity	as recorded at the non-EU migrant pre-entry tuberculous screening or refugee pre-entry health assessment
Visa category	as recorded at the non-EU migrant pre-entry tuberculous screening or refugee pre-entry health assessment
Country of origin	as recorded at the non-EU migrant pre-entry tuberculous screening or refugee pre-entry health assessment
Country of departure	as recorded at the refugee pre-entry health assessment
Date of pre-entry tuberculous screening or refugee pre-entry health assessment	as recorded at the non-EU migrant pre-entry tuberculous screening or refugee pre-entry health assessment
Length of time in England prior to index admission/death	(in years) at the given time point will be estimated as (date of admission/death - month & year of pre-entry screening/365.25)
Age at index admission/death	(in years) at a given time point will be estimated as ((date of admission/ death – month & year of birth)/365.25) for the index admission or death
ICD-10 chronic disease conditions	obtained from all admissions at the index admission (sub-divided into categories of: 1. mental health/behavioural 2. cancer/blood disorders 3. chronic infections 4. respiratory 5. metabolic/endocrine/nutritional 6. renal/genitourinary 7. musculoskeletal/dermatological 8. neurological, 9. cardiovascular

### Sensitivity analyses

To determine the robustness of our final results and to quantitatively account for any uncertainty, sensitivity analyses will be conducted to examine the extent to which our findings are affected by changes in methods or values of unmeasured variables. We are uncertain about the length of time non-EU migrants remain in England following their arrival. In a first sensitivity analysis, all migrants will be assumed to stay for one and a half years, the median time of stay for an international migrant, providing a lower estimate of person time at risk. In a second sensitivity analysis, all migrants will be assumed to stay until the end of the study period of 31st December 2017. This is the more conservative assumption, and whilst it unrealistically inflates the denominator, it provides a lower bound for the estimates of incidence and prevalence.

### Dissemination of results

At the end of the study we will convene a group of policy makers, non-governmental organizations, migrants and refugees and the public to feedback the results of our study and seek suggestions on ways to take this work forwards. We will also disseminate our findings to relevant policy makers and schemes through a series of regional workshops.

### Study status

We are currently in the process of seeking ethical and Confidentiality Advisory Group (CAG) approvals for the study.

## Discussion

We describe a novel record linkage study that will use routine data from multiple sources to generate the Million Migrant study. The study has several advantages including the opportunity to accurately identify migrants in UK routine health records and their subsequent health needs. The strengths of this approach are the creation of a highly-powered cohort study that harnesses existing data to uncover health patterns in this often difficult to identify or invisible population. The Million Migrant study aims to improve evidence on hospital-based events and mortality and will better position the scientific community to inform policy makers and civil society with rigorous data about the health of migrants in England.

There are several limitations to our study. We will be unable to include data from primary care due to the lack of a national dataset available for this purpose. As a result, we will be unable to examine any contribution to the health and care of the individuals from primary and community or social care. Designing a new linkage of the million migrant cohort to primary care data would provide a more comprehensive understanding of primary care usage within this population and allow us to identify new opportunities for community-level interventions, and this may become a possibility in the coming years. We also do not include data from all migrant sub-groups. Irregular migrants (e.g. entrants who enter, stay or work in a country without the necessary authorization such as undocumented entrants, failed asylum seekers, visa overstayers, children born to irregular migrant couples), migrants entering on a temporary visa (e.g. tourist visa), EU and EEA migrants, international migrants from low-incidence tuberculosis countries who subsequently do not have a pre-entry tuberculous screening (e.g. United States of America, Chile, and Egypt), and international migrants who emigrated before the start of either health screening programme will not be captured. As such, the study findings will not be generalisable to these groups. Additionally, although smaller in number, these groups could potentially be included in our randomly generated sample from the general population residing in England. This is important to consider in our interpretation of the findings. Our proposed study will not be able to assess whether the healthcare and mortality outcomes were affected by frequency of travel abroad, health service usage abroad, uncertainty in length of residence in the UK, movement in the UK outside of England to access healthcare, or wider socio-environmental determinants of health. These factors will be later examined through the creation of an electronic longitudinal cohort study using a mobile phone application to collect data on the health of migrants after moving to the UK (part of RWA’s Wellcome Trust Fellowship, Public health data science to investigate and improve migrant health in the UK).

To help ensure impact from the work, we have engaged with policy makers, non-governmental organizations, migrants and refugees and the public throughout the design and conduct of the study to ensure relevance to them and prepare a pathway for impact, and will continue to do this through to the end of the project. In designing this study, we held a workshop with international migrants and refugees to understand their views on the consent process, data linkage and analysis. We have also involved national and international policy makers in the design stage.

In England today, nearly 15.6% of the population are international migrants, constituting an important and large group of people. However, there is still a limited understanding of their health needs and use of secondary care in the NHS. This study will fill an important gap in the literature and provide local, regional and national policy makers with detailed information about the health needs of this population. Our results will include information about how and where healthcare services can be improved to prevent hospital admissions, and data on the causes of death in this large and important group of people. Whilst maintaining the highest scientific and ethical standards on the use of existing data, or big data, we will set forth an avenue to advance knowledge and good practices in the field of migration and health, for the UK and internationally.

## Data availability

### Underlying data

No data is associated with this article.

### Extended data

All extended data is publicly available on Open Science Framework: Million Migrants study of healthcare and mortality outcomes in non-EU migrants and refugees to England: Analysis protocol for a linked population-based cohort study of 1.5 million migrants, https://doi.org/10.17605/OSF.IO/FUTS4 ^15^

Files available:

- Extended data 1: Tuberculosis pre-entry screening rollout phases
- Extended data 2: Revised definition of avoidable conditions
- Extended data 3: Avoidable conditions definition for children and young people
- Extended data 4: Definition of Ambulatory Care Sensitive Conditions
- Extended data 5: Definition of sexual and reproductive health outcomes
- Extended data 6: Definition of mental and behavioural disorders
- Extended data 7: Deduplication of records
- Extended data 8: RECORD checklist

Data are available under the terms of the Creative Commons Zero "No rights reserved" data waiver (CC0 1.0 Public domain dedication).

### Reporting guidelines

To review the study’s RECORD checklist, please see Extended data file 8.
