# Supplementary material for: Million Migrants study of healthcare and mortality outcomes in non-EU migrants and refugees to England: Analysis protocol for a linked population-based cohort study of 1.5 million migrants
Source: Wellcome Open Res. 2019 Jan 17;4:4. [Version 1] doi: 10.12688/wellcomeopenres.15007.1 (PMC6381442; doi:10.12688/wellcomeopenres.15007.1)
Supplement: Peer review history

# Peer review: Reviewer response for version 1

**DOI:** 10.21956/wellcomeopenres.16372.r34598

**Published:** 2019-03-04

**Authors:** Ursula Trummer

**Affiliations:** 1 Center for Health and Migration, Vienna, Austria

The authors do present an interesting paper on a timely subject. I will not duplicate scope and purpose or data sets used, as this is outlined in detail by the authors.

 The paper reads very technical, and addresses an inner circle of scholars. A potential audience outside this inner circle may be lost after the first paragraph or the abstract.

 Although it presents and refers to the conceptual framework for influences on migration and health adapted from the UCL Lancet Commission on migration and health, a link from this conceptual framework to the actual analysis is presented only to a marginal degree. In some lines the text presents a rather restricted causal approach of “use of health services and subsequent health conditions” (in the abstract and the introduction paragraph) which may suggest to the reader that the use of health service is the main determinant of migrant’s health conditions.

 The analyses include some interesting outcome subgroups, suitable for further secondary analysis e.g. for economic considerations, like ambulatory sensitive conditions (ACS), amendable conditions, preventable conditions. It would be interesting to know if the data will be made available to other researchers for such secondary analysis purposes.

I have read this submission. I believe that I have an appropriate level of expertise to confirm that it is of an acceptable scientific standard.

# Peer review: Reviewer response for version 1

**DOI:** 10.21956/wellcomeopenres.16372.r34600

**Published:** 2019-02-15

**Authors:** Sarah Rodgers

**Affiliations:** 1 Health Data Research UK (HDR-UK), Swansea University, Swansea, UK; 2 Department of Public Health and Policy, University of Liverpool, Liverpool, UK

The paper sets out a protocol to investigate non-EU migrant and refugee health care utilisation and mortality against that of the host country, the UK. The aim is to investigate rates to understand if there are barriers for migrants accessing healthcare. For instance they mention how only a third of migrants are registered with a GP.

 It describes their intention to use routinely collected data for a subset of the total migrant population. Those who receive a refugee health check. And those who are screened for TB because they come from originating countries with high TB rates. It is not possible to analyse all migrants; only those who enter as refugees and those who undergo screening because they originate from countries with high TB rates. This is the basis of their migrant cohort, which, as authors point out, omits migrants from developed countries with few TB cases.

 Data linkage

 I concentrate here on data linkage aspects because two reviewers have previously covered other aspects. The data linkage relies on the Personal Demographics Service (PDS) dataset. This dataset is key because they will use it to add NHS numbers to the migrant/refugee datasets to add NHS numbers and enable deterministic joining using the NHS number of the migrant cohort to their corresponding records in HES and ONS mortality datasets.

 While there is an extended file on the de-duplication of refugee/migrants to create the migrant cohort, there are not additional files on the linkage. I suggest one is added. As it currently stands, there are insufficient details in the methods to allow replication. The datasets they will be using would have some useful additional detail. Their explanation would benefit from details: What is the percentage of NHS numbers added by PHE? What will they do with the cohort members who do not have an NHS number? The completeness of the PDS dataset, and allocation of an NHS number relies on the person having utilised health care services. What is the percentage of the cohort they expect to have an NHS number?

 What will they do if the PDS contains only a third of the migrants who have registered with a GP, as highlighted by Stagg et al. ^1^? I remain sceptical about the PDS having data added through all the sources mentioned on the NHS Digital website (pharmacies, child and mental health, secondary care providers) and believe the main source of data is from the patients themselves when they register with a new GP. Checking thoroughly with NHS Digital at this stage may save time later. Or adding such reassurances to the protocol would help.

 Will they simply assume no NHS number means no utilisation? Thereby including these cohort migrants in the no healthcare utilisation denominator? They are using data up until end 2017 and it is likely the rates of NHS numbers populated increase the longer they spend in the UK. Adding time since entry to the UK would also be a useful variable to stratify your comparisons. A young healthy migrant is unlikely to need any healthcare, particularly if they are male, due to lack of screening and reproductive care needs and I note they will use sex in comparing matching levels.

 Outcomes and analysis plan

 I suggest that unplanned (emergency) admissions are the type that should be avoided ideally, and are also an indicator that there is a lack of preventative care. Can this be added as an additional group for analysis? These then would be separate from the planned admissions that have a prevention goal.

 There seems like there is an opportunity to model several outcomes together in a combined model, for instance using multivariate generalized linear mixed models. This would place the relative importance of the different outcomes in the same model and take into account multiple testing corrections they will have to complete otherwise (e.g. Bonferroni). Therefore they should use a smaller significance level to account for multiple hypothesis testing; there are four outcomes for healthcare utilisation, for instance. Justification of the use of Cox regression seems a little confused. They should rephrase as time to event (e.g. hospital attendance) rather than specify the follow up time as the event itself. Their note about confirming statistical modelling approaches following review of data is usual in the case of routine data when the distribution of each outcome variable is unknown. Why not use Cox regression for the all cause death outcome also? Overall their analysis plan seems underspecified.

 Sample size

 I note that they mention loss of 10% from migration to Scotland and Northern Ireland. Although the proportion moving to Wales will be small, it is incorrect to include Wales because these data are held in the Patient Episode Dataset for Wales (PEDW) and are not included in the HES dataset. This needs adjusting.

 Discussion/additional points

 I agree with the first reviewer that it is particularly important the authors remove the conflicting statements about the analytical potential to examine the wider determinants of health.

I have read this submission. I believe that I have an appropriate level of expertise to confirm that it is of an acceptable scientific standard, however I have significant reservations, as outlined above.

## References

[1] StaggHRJonesJBicklerGAbubakarI: Poor uptake of primary healthcare registration among recent entrants to the UK: a retrospective cohort study. *BMJ Open*.2012;2(4) : 10.1136/bmjopen-2012-001453 10.1136/bmjopen-2012-001453 22869094PMC4400681

# Peer review: Reviewer response for version 1

**DOI:** 10.21956/wellcomeopenres.16372.r34599

**Published:** 2019-02-07

**Authors:** Matthew Wallace

**Affiliations:** 1 Stockholm University Demography Unit (SUDA), Stockholm, Sweden; 2 French National Demographic Institute (INED), Paris, France

**Summary**

 This article summarizes the analysis protocol for a study investigating refugees and migrants originating from non-EU countries with a high prevalence of tuberculosis in England. Specifically, the authors plan to link four data sources to generate a population-based cohort study of around 1,3 million people. The study will examine hospital-based events and all-cause and cause-specific mortality among (specific groups of) migrants in England in the past decade. The study will not be representative of international immigration to England because EU-migrants and non-EU migrants from countries with low tuberculosis prevalence cannot be identified (and are in fact grouped with the English-born as part of the reference population). This is not problematic (definition of the reference group aside) because the biggest value in this study lies in the ability to identify and study such outcomes among *refugees*, so long as the data is packaged in such a way. Previous studies into the health and mortality of refugees in England are lacking because of an inability to identify them in national data sources. However, the authors should recognize that this is not the case for international immigrants in England in general, which is the impression they give in the introduction. Additionally, in some areas of the protocol the authors could be more explicit (e.g. with regards to potential errors induced in how the entries and exits of migrants are captured). Nonetheless, this large-scale study has a great deal of potential and could provide significant insight into health and mortality of refugees and non-EU migrants from countries with high tuberculosis prevalence, in turn contributing to the existing body of literature on the migrant mortality advantage in England.

**Introduction**

 In the introduction, the authors tell the readers that little research has been conducted on mortality among international migrants in England. While this is (I think) accurate for access to health services and health, this is less true for immigrant mortality. See references provided. Together these studies document variation among international immigrants with respect to their all-cause and cause-specific mortality, country or region of origin, generation, age, sex, and duration of residence (among other factors). It is important to give appropriate acknowledgement to previous works so as not to *appear* to be overstating the gap in current literature in England. Acknowledging these works would not lessen the contribution of this study. There are limitations in these studies (including my own) upon which the “Million Migrants study” can build to provide exciting new insight into migrant mortality, health, and health care access patterns in England.

 Additionally, it is important to note that other national data sources exist in which one can identify migrants and examine these outcomes, contrary to that suggested by the authors. For example, see the *Office for National Statistics Longitudinal Study *(LS) which combines data from National Health Service systems, censuses and civil registers. With the LS data, it is possible to identify migrants and examine all-cause and cause-specific mortality, cancer registrations, self-reported health and long-term illness.

 The above issues stem from how the study is framed: around all international immigrants in England. Indeed, the introduction defines international migrants in the broadest possible terms. However, the Million Migrants Study excludes a significant proportion of all international immigrants (at least 40%). With the study focusing mainly on refugees and non-EU migrants from high TB prevalence countries, the introduction should explicitly focus on these groups. Then it *would* be accurate to state that there is a lack of studies in England on refugees (I think just one, see: Swerdlow, 1991 ^1^). Similarly, then it *would* be accurate to state that there is a lack of national data sources in England that have the relevant information to be able to identify refugees (which would require information on country of birth, reason for arrival, visa type etc…). I recommend the authors re-frame their introduction around the migrant subgroups they are studying, place their study more specifically in the context of the previous literature on migrant mortality in England ^1^^-^^14^, and be more explicit in their phrasing.

**Aims**

 As alluded to above, the study sample is selective: refugees and non-EU migrants from countries with high tuberculosis prevalence. This is fine and explicitly acknowledged by the authors in their discussion as a limitation. However, it conflicts with a repeatedly-stated objective to investigate whether or not a migrant mortality advantage can be replicated in England (this also taps in to how the introduction is framed). Of course, a migrant mortality advantage has already been observed in England (and Wales). Observing, or indeed not observing, lower mortality among the migrants in this sample relative to the general population would not provide evidence of an overall migrant mortality advantage in England. Suggest the authors qualify such statements to reflect this.

**Data collection, processing, and linkage**

 Are data on EU migrants simply not available or is it a choice by the authors to exclude them? If the data is not available, then the authors can simply state this. Better to do this than to over-generalize about culture and use of health care systems among EU migrants. If it is a restriction of the data, the exclusion does not need to be further justified and cannot be avoided. If, on the other hand, it was a choice, then the authors should consider including EU migrants to provide a broader appeal of this new data source for its potential users.

 Will the study cover immigrants arriving at all ages?

 Exactly what variables will be available in this data source? It might be nice if the authors were to provide a preliminary list to give some indication of the scope of the data.

**Comparator groups**

 It is not possible to remove other types of migrants who did not undergo screening from the general population? Further, it is important to note that the general population will include children of migrants, who form a small but growing proportion of England’s population. A growing body of research suggest that children of migrants have different mortality patterns to majority populations of high-income host countries too. As a basic approximation, if we take the 6,4% of EU migrants, some proportion of the 9,2% of non-EU migrants who are not subjected to screening, and the 9,2% of the population who are children of migrants (from Eurostat, 2014), then approximately 16% of the general population is formed of individuals who should be excluded (or form other categories). Is there any additional information (country of birth or ethnicity) the authors can use to remove these individuals from the general population?

 More information on exactly how the general population is sampled would also be very useful. Knowing this is *as important* as knowing migrants are accurately defined.

 Please expand upon the rationale behind comparing migrants, who have just arrived and have very little exposure to conditions in England, to the general population by IMD score.

**Entry and exit from cohort**

 Accurately calculating exposures for mobile populations such as migrants is one of the most challenging tasks to achieve in the studies of migrant populations (see previous studies on under-coverage, over-coverage, censoring bias etc…). With this in mind, it would be informative if the authors devoted a little more space to telling us exactly how immigrant entries and exits are captured in their data and discussing the potential biases incurred from the information they have used.

 For example, entries are defined as the latest date of two pre-entry screenings  Could this lead to cases where individuals are considered “at risk” when they are still living in their origin countries? Are delays between pre-entry screening and arrival to England common? How long do they tend to be? Are there people who undertake screening, but never arrive in England? What happens to these individuals? Could they be incorrectly included in the data source?

 Some clarification on the way emigrations are imputed would also be helpful (rather than linking to previous studies). Is there any information in the four data sources that could provide some (in)direct information on (r)emigration? These events are unlikely to be negligible among foreign-born and a lack of knowledge about exactly when people leave can contribute to the downward biasing of mortality rates. I appreciate the sensitivity analysis that the authors have done in response to this issue. Nonetheless, a good deal of uncertainty remains around when exactly individuals leave the country.

**Sample size**

 I am not sure if the sample size of the general population is stated anywhere (forgive me if I missed it). We know that the study will contain around 1.3 million migrants. It would make sense to state the general population figure here too to provide overall sample size.

I have read this submission. I believe that I have an appropriate level of expertise to confirm that it is of an acceptable scientific standard, however I have significant reservations, as outlined above.

## References

[1] SwerdlowAj: Mortality and cancer incidence in Vietnamese refugees in England and Wales: a follow-up study. *Int J Epidemiol*.1991;20(1) :13-9 206621010.1093/ije/20.1.13

[2] GuillotMKhlatMEloISolignacMWallaceM: Understanding age variations in the migrant mortality advantage: An international comparative perspective. *PLoS One*.2018;13(6) : 10.1371/journal.pone.0199669 e0199669 10.1371/journal.pone.0199669 29958274PMC6025872

[3] HardingS: Mortality of migrants from the Caribbean to England and Wales: effect of duration of residence. *Int J Epidemiol*.2004;33(2) : 10.1093/ije/dyh059 382-6 10.1093/ije/dyh059 15082645

[4] HardingSRosatoMTeyhanA: Trends for coronary heart disease and stroke mortality among migrants in England and Wales, 1979-2003: slow declines notable for some groups. *Heart*.2008;94(4) : 10.1136/hrt.2007.122044 463-70 10.1136/hrt.2007.122044 17690159PMC2565582

[5] HardingS: Mortality of migrants from the Indian subcontinent to England and Wales: effect of duration of residence. *Epidemiology*.2003;14(3) :287-92 12859028

[6] HardingSBalarajanR: Patterns of mortality in second generation Irish living in England and Wales: longitudinal study. *BMJ*.1996;312(7043) :1389-92 864609510.1136/bmj.312.7043.1389PMC2351124

[7] HardingSBalarajanR: Mortality of third generation Irish people living in England and Wales: longitudinal study. *BMJ*.2001;322(7284) :466-7 1122242210.1136/bmj.322.7284.466PMC26563

[8] ScottAPTimæusIM: Mortality differentials 1991-2005 by self-reported ethnicity: findings from the ONS Longitudinal Study. *J Epidemiol Community Health*.2013;67(9) : 10.1136/jech-2012-202265 743-50 10.1136/jech-2012-202265 23740930

[9] WallaceM: Adult mortality among the descendants of immigrants in England and Wales: does amigrant mortality advantage persist beyond the first generation?. *Journal of Ethnic and Migration Studies*.2016;42(9) : 10.1080/1369183X.2015.1131973 1558-1577 10.1080/1369183X.2015.1131973

[10] WallaceMKuluH: Low immigrant mortality in England and Wales: a data artefact?. *Soc Sci Med*.2014;120: 10.1016/j.socscimed.2014.08.032 100-9 10.1016/j.socscimed.2014.08.032 25233336

[11] WallaceMKuluH: Mortality among immigrants in England and Wales by major causes of death, 1971-2012: A longitudinal analysis of register-based data. *Soc Sci Med*.2015;147: 10.1016/j.socscimed.2015.10.060 209-21 10.1016/j.socscimed.2015.10.060 26595089

[12] WallaceMKuluH: Can the salmon bias effect explain the migrant mortality advantage in England and Wales?. *Population, Space and Place*.2018;24(8) : 10.1002/psp.2146 10.1002/psp.2146

[13] WildSHFischbacherCBrockAGriffithsCBhopalR: Mortality from all causes and circulatory disease by country of birth in England and Wales 2001-2003. *J Public Health (Oxf)*.2007;29(2) : 10.1093/pubmed/fdm010 191-8 10.1093/pubmed/fdm010 17456532

[14] WildSHFischbacherCMBrockAGriffithsCBhopalR: Mortality from all cancers and lung, colorectal, breast and prostate cancer by country of birth in England and Wales, 2001-2003. *Br J Cancer*.2006;94(7) : 10.1038/sj.bjc.6603031 1079-85 10.1038/sj.bjc.6603031 16523198PMC2361230

# Peer review: Reviewer response for version 1

**DOI:** 10.21956/wellcomeopenres.16372.r34597

**Published:** 2019-01-25

**Authors:** Jessica L. Potter

**Affiliations:** 1 Centre for Primary Care and Public Health, Queen Mary University London, London, UK

This article summarises the analysis protocol for a linked population cohort study.  The aim of the study is to describe hospital-based healthcare and mortality outcomes among 1.5 million non-EU migrants.  This is because immigration status is not routinely collected on a large scale in the UK to allow comparative studies of health outcomes between migrant populations and UK-born populations.  

 The research team plan to compare outcomes between migrants, refugees and the general population of England.  To do this they will collate three sources of data: 1. Data from pre-entry screening of non-EU migrants from high burden TB countries 2. Pre-departure health screening of refugees. 3 National hospital episode statistics. 4. ONS death records.

 The authors clearly identify a gap in knowledge with respect to migrant health and therefore this large study could provide extremely novel and valuable insights.

 Protocol:

 To justify the focus on non-EU migrants the authors state, "We focus on non-EU migrants because of their availability in this dataset, but also because EU migrants are more likely to dual-use health systems and less likely to need adaptation in terms of cultural competence ^13^" 

 - I have reviewed the paper referenced.  It is a systematic review which compiled predominantly qualitative studies thus I could not find evidence to support the claim that "EU migrants are more likely" to dual-use health systems.  Equally, I think the claim that EU migrants are less likely to need adaptation in terms of cultural competance essentialises the experience of being an EU migrant.  Equally, not all aspects of an individuals culture play an equal role in determining how they access care.  I suggest that the authors simply make the statement that the focus on non-EU migrants is because of the availability of the data set.  Similarly, I think that it should be made very clear that it is not simply a focus on non-EU migrants but non-EU migrants from a very specific cohort of 101 countries, the majority of which are LMIC. Given pre-entry screening for TB is performed in high burden countries and TB burden at country level has been shown to be related to GDP which in turn is related to mortality, does this not introduce a selection bias at the beginning? I realise this is mentioned in the limitations section of the discussion but I think it would be useful to comment on the different characteristics of the groups not included and how these might affect the study outcomes.

 I also think it should be made clear that not all refugees are screened pre-departure, some individuals claim asylum on arrival in the UK prior to becoming refugees.  Please could the authors make a comment on the representativeness of this cohort of refugees in relation to the wider refugee population in the UK?

 Ethics

 I note in figure 2 (if I have interpreted it correctly) a plan to share identifiable data between Public Health England and NHS Digital.  I wondered whether the authors have sought assurances that such data will not be shared with other parts of government for the purposes of immigration control as has previously occurred through NHS Digital.  Beyond this concern the authors have clearly outlined a robust plan of psuedoanonymisation.  

 Comparator groups

 The authors state the general population of England will be "composed of mostly England-born residents".  Is country of birth routinely collected to allow this assumption or will the size/ methods of selecting this matched sample ensure they represent the background England population which the authors point out later in the paper consists of 15% of individuals originally born outside the UK?

 Analysis plan

 The authors state in the introduction "the study includes analyses that acknowledge the wider determinants influencing the health of international migrants such as the legal, social, economic and health structures and systems, health service access and support, exposures and behaviours, and epidemiological changes associated with population mobility" but no details of how this will be done are provided. Then in the discussion the authors state they will be unable to assess the impact of the "wider socio-environmental determinants of health" - please could they therefore clarify the earlier point as I think it is a little mis-leading as it stands.

 I cannot comment on the details of the mathematical aspects of the analysis plan as this is outside the range of my expertise.

I have read this submission. I believe that I have an appropriate level of expertise to confirm that it is of an acceptable scientific standard.
